# Zebrafish structural development in Mueller-matrix scanning microscopy

**DOI:** 10.1038/s41598-019-56610-9

**Published:** 2019-12-27

**Authors:** Aymeric Le Gratiet, Marta d’Amora, Marti Duocastella, Riccardo Marongiu, Artemi Bendandi, Silvia Giordani, Paolo Bianchini, Alberto Diaspro

**Affiliations:** 1 Nanoscopy and NIC@IIT, Istituto Italiano di Tecnologia, Via Morego 30, 16163 Genoa, Italy; 2 Nano Carbon Materials, Istituto Italiano di Tecnologia, Via Morego 30, 16163 Genoa, Italy; 3 Nanophysics, Istituto Italiano di Tecnologia, Via Morego 30, 16163 Genoa, Italy; 4 Department of Physics, University of Genoa, Via Dodecaneso 33, 16146 Genoa, Italy; 5 School of Chemical Sciences, Dublin City University, Glasnevin, Dublin 9, Ireland

**Keywords:** Embryology, Polarization microscopy

## Abstract

Zebrafish are powerful animal models for understanding biological processes and the molecular mechanisms involved in different human diseases. Advanced optical techniques based on fluorescence microscopy have become the main imaging method to characterize the development of these organisms at the microscopic level. However, the need for fluorescence probes and the consequent high light doses required to excite fluorophores can affect the biological process under observation including modification of metabolic function or phototoxicity. Here, without using any labels, we propose an implementation of a Mueller-matrix polarimeter into a commercial optical scanning microscope to characterize the polarimetric transformation of zebrafish preserved at different embryonic developmental stages. By combining the full polarimetric measurements with statistical analysis of the Lu and Chipman mathematical decomposition, we demonstrate that it is possible to quantify the structural changes of the biological organization of fixed zebrafish embryos and larvae at the cellular scale. This convenient implementation, with low light intensity requirement and cheap price, coupled with the quantitative nature of Mueller-matrix formalism, can pave the way for a better understanding of developmental biology, in which label-free techniques become a standard tool to study organisms.

## Introduction

Over the last decades, zebrafish (*Danio Rerio*) have become established animal models for understanding the development of several human pathologies. The structural organization of zebrafish presents numerous similarities with the anatomy and physiology of other higher-order vertebrates, including humans^1^. Furthermore, zebrafish have a high-reproductive rate and offer excellent conditions for deep imaging thanks to their high optical transparency and small size at the embryonic and larva stages^2^. Advanced optical microscopy techniques, such as confocal or light-sheet microscopy, are normally employed to obtain information on the structural organization of these models at the single cellular level^3^. The main advantage of such a technique is allowing the specific and sensitive labeling of sample to detect localized features or molecules of interest under illumination. These microscope architectures provide real-time subcellular structural data of live zebrafish at early developmental stages, but they impose certain restrictions on the sample characteristics or on the sample preparation. Firstly, the embryo and larvae can have intrinsic scattering signatures that limit light penetration on the overall thickness of the sample. Secondly, the high transparency of embryos/larvae in the optical wavelengths typically leads to low contrast, resulting in images with a low Signal-to-Noise Ratio (SNR). Adding fluorescence labels can solve this, but exogenous contrast agents can interfere with the natural physiological processes of the animal. Additionally, the preparation of the fluorescence labeling is a mandatory and time-consuming step that can depend on the particular labeling protocol used.

Label-free microscopy techniques can overcome these issues and advance towards the imaging, with high contrast, of thick transparent zebrafish specimens at different embryonic stages. To date, only a few techniques, including the combination between Second Harmonic Generation (SHG) imaging and polarization detection, known as polarization resolved SHG (pSHG), have been used to characterize these animal models^4–6^. Even if the quantitative cellular orientation of the fibrillary structures of zebrafish have been reported with pSHG, these methods require complex and expensive setups and high laser intensities, which can induce irreversible damage to the sample at these early stages. Furthermore, the information available is limited to only a few polarimetric effects, such as the birefringence present in every biological organism. For this reason, an easy and cheap method to characterize the birefringence and its orientation of the muscle tissue is based on simple crossed-polarization imaging^7–10^. However, the contrast and resolution depends strongly on the orientation of the sample, limiting the interest of such a technique.

Mueller-matrix microscopy is an alternative label-free technique providing a complete characterization of the polarization signature of a sample. The fundamental principle of the microscope consists of generating well-known polarization states and analyzing the transformation after interaction with the sample^11^. At least 16 intensities are needed to completely determine the 4 × 4 Mueller-matrix of the sample, where each independent elements named m_ij_, can be related to a specific physical phenomenon, such as linear or circular dichroism, birefringence, and scattering^12–14^. The physical model to quantify all the polarimetric effects characterizing the structural organization of the non-labeled sample is an important issue and can be achieved by decomposing mathematically the Mueller-matrix with the most common approach, which is the Lu and Chipman decomposition^11,15–17^. While Mueller-matrix microscopy has been successfully demonstrated in numerous research fields, such as materials science^18^, ophthalmology^19,20^, biomedical diagnosis^21–27^ and both transmission and reflection multimodal microscopy imaging^28–30^, its usage in developmental embryology has yet to be reported^31^. The main reason is that the polarimetric signature of that sample is difficult to understand compared to materials sample, and that is why these label-free methods are not typically used in biology.

Here, we show how a home-built Mueller-matrix scanning microscope in reflection configuration enables the quantitative characterization of the structural organization of fixed and preserved zebrafish at different development stages. In this work, we analyzed the statistical distribution of the polarimetric contrast of the whole fish during the development, including the fibrillary organization (dichroism), the multi-structural orientation (phase, birefringence), and thickness inhomogeneity (scattering). As our results demonstrate, the Lu Chipman decomposition enables correlating the contrast results with different parts of the zebrafish specifically, which would not be possible with other conventional label-free microscopy techniques.

## Results

### Developmental stages under bright field imaging

**Figure 1:**
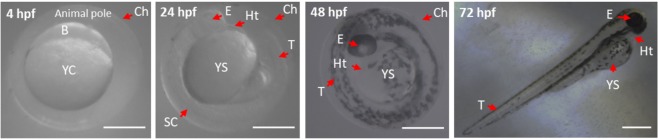
Optical bright field images of fixed zebrafish embryos and larvae at different stages of development. At 4 hpf (hours post fertilization) (blastula stage), the embryo is formed by yolk cells (YC) and blastoderm (B). Moreover, a protective barrier, named chorion (Ch), surrounded the embryo. At 24 hpf (pharyngula stage), it is possible to identify the heart (Ht), eyes (E), spinal cord (SC) and the tail (T) attached to the yolk sac (YS). At 48 hpf, the embryo has a well-structured spine and evident skin pigmentation. At 72 hpf (larval stage), the embryogenesis is completed and the pigmentation progressive increased dorsally. The scale bars correspond to 500 μm.

Figure 1 shows a bright field image for each developmental stages, which are in good agreement with those expected from the typical zebrafish development^32^. Due to their small size, these first developmental stages are good candidates to characterize the structural changes of biological tissues, which can be fully imaged within the field-of-view of a 4X/0.1NA objective. Moreover, each sample is around 0.5–1 mm thickness and composed mainly of water, thus limiting the polarimetric artifacts through the double-pass interaction with the whole sample.

### Developmental stages under Mueller-matrix imaging

**Figure 2:**
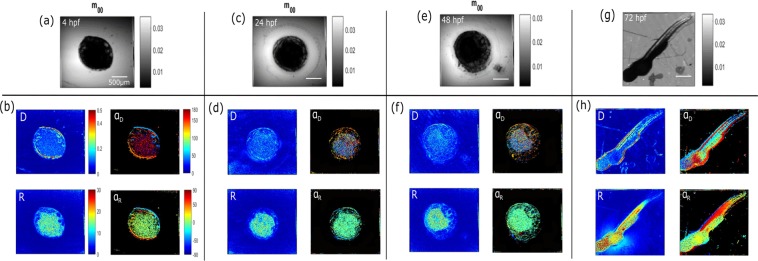
Polarimetric images of fixed zebrafish embryos and larvae at 4 hpf, 24 hpf, 48 hpf and 72 hpf using a 4X/0.1NA objective. The images (**a**),(**c**),(**e**),(**g**) correspond to the total collected intensity from the Mueller-matrix, which is the m_00_ element. The images (**b**),(**d**),(**f**),(**h**) are the Lu and Chipman decomposition images, i.e coded in Diattenuation (D), in Retardance and its azimuthal orientations α_D_ and α_R_, respectively.

Figure 2 presents the images acquired using a 4X/0.1NA objective coded in total collected intensity (m_00_) and in polarimetric parameters after applying the Lu and Chipman decomposition, corresponding to the diattenuation (D), the retardation (R) and the respective azimuthal orientation (α_D_ and α_R_). As expected, the m_00_ images from Fig. 2 display the same features as those acquired with a bright-field microscope (Fig. 1). Nevertheless, at 4 hpf, 24 hpf, and 48 hpf, the attenuation was stronger for the yolk cell/sac (YC/YS) area, due to the double-pass interaction through the whole thickness, which reduced the quantity of collected light compared to Fig. 1. After 48hpf, the eyes and the tail could be distinguished with images showing an enhanced contrast for the different tissues that compose the embryo. The full potential of the Mueller-matrix microscope comes when analyzing the four polarimetric images after the Lu and Chipman decomposition. Regarding the images of the diattenuation D, they indicate the linear dichroism effects induced by the fibers of the sample. As shown in Fig. 2(b),(d),(f),(h), at each stage of development, the embryo exhibits a value of D between 0.1 and 0.4. Interestingly, at 4hpf we can distinguish a high contrast between the animal and vegetal poles. This can be explained by the weak attenuation of the zebrafish composed mainly of thin and transparent biological structures, including the chorion and the blastoderm, while the attenuation becomes stronger for the yolk cell. The values of the fiber orientation through the development process represented by α_D_ display, in all cases, higher contrast than D. Indeed, the development of increasingly complex structures, such as the blastoderm, offers an increased contrast. This is also the case for the larvae at 72hpf, which allows one to discern the cartilage, the blood vessels and part of the tail muscles. Concerning the R values, they are influenced by the refractive index changes of the zebrafish. At the first development stage, the structure of the embryo is compact, thus inducing strong phase effects on the polarized light. As the embryo evolves and becomes more complex, the compactness of its structure decreases, thus resulting in locally smaller and less spatially homogeneous R values. This can be observed when looking at the values for newly created structures, such as the head or the eyes. Instead, the yolk sac, which is the only structure expected to be present at every stage of the embryo, shows a constant R value during development. This loss of birefringence contrast is not an issue by comparing it with the images corresponding to the retardance orientation α_R_. Indeed, this parameter provides a more localized contrast for every structure of the embryonic stages, such as the eye and the cartilage fibers. Furthermore, contrary to the three other polarimetric coded images, the α_R_ image at the larvae stage gives access to the precise location of the tail muscle fibers. Note that the chorion could not be distinguished in the Lu and Chipman decomposition images due to the thin thickness of this lipid part, which induced weak polarimetric effects relating to the yolk sac region.

### Statistical analysis of the polarimetric changes

**Figure 3:**
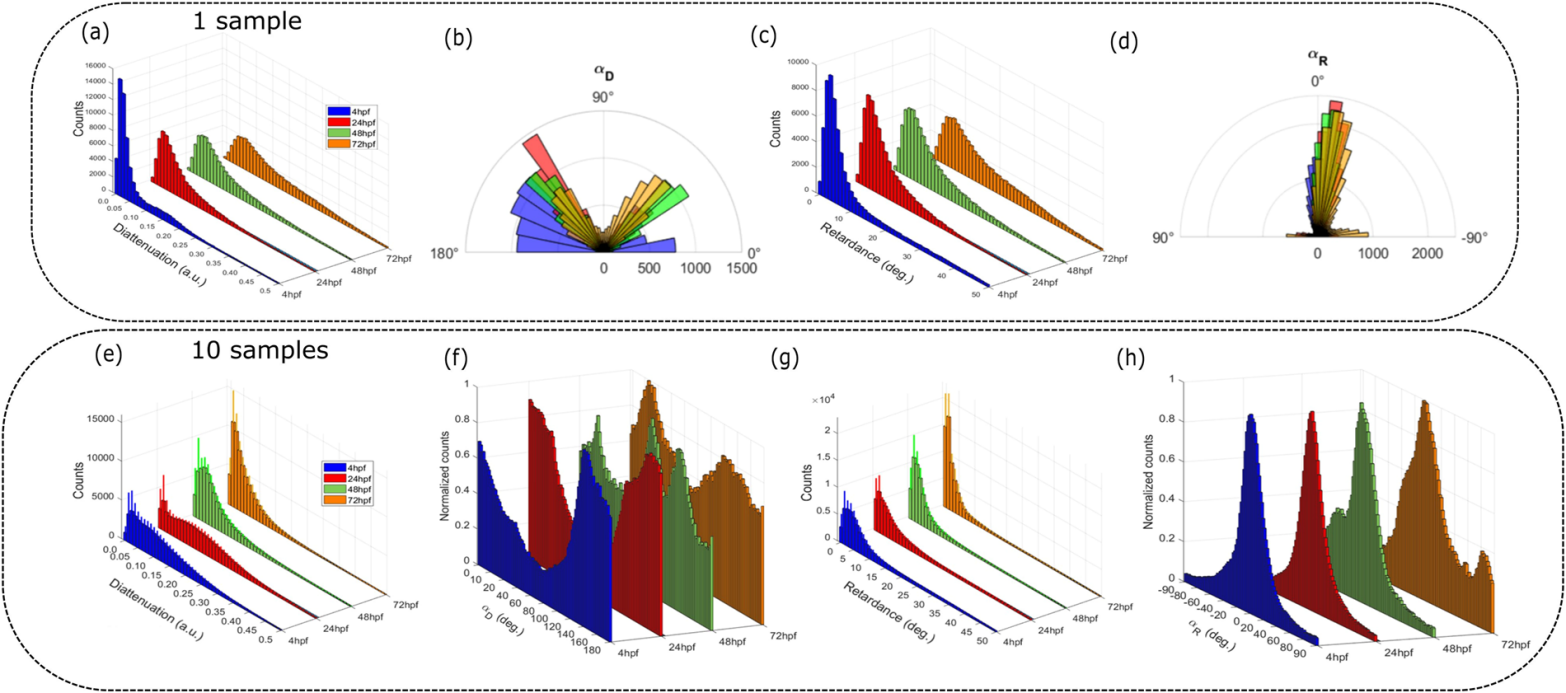
(**a–d**) Histograms of the pixel values of the four polarimetric parameters at each development stage from Fig. 2 (one sample). (**e–h**) Averaged histograms over 10 samples of the four polarimetric parameters at each development stage. The blue, red and green and orange plots corresponded to 4 hpf, 24 hpf, 48 hpf and 72 hpf respectively.

The pixel distribution histograms of the previous images provide a quantitative description of the polarimetric effects during zebrafish growth (Fig. 3(a)–(d)). In order to characterize the statistical polarimetric trend, we acquired images of 10 samples at each stage (Fig. 3(e)–(h)). A clear distinction for the diattenuation distribution can be made between the first and last developmental stages. Indeed, the more compact structures at 4 hpf and 24 hpf present a wider distribution of the D values since the embryo is not biologically well defined. On the other hand, the two last stages presented a narrow distribution of D values, thus indicating localized structural changes during growth. A more detailed evolution of the morphological changes experienced by the zebrafish between 4 hpf and 72 hpf can be seen in the changes of the azimuthal orientation α_D_. In fact, the embryo at 4 hpf presents values mostly around 140°, while the larvae at 72 hpf present two main orthogonal orientations at around 50° and 130°, surrounded by all the possible angles. This is explained by the complexification of the fibrillary structure for the newly developed organs. Between these two stages, the orientation distribution observed at 24 hpf and 48 hpf at these two orthogonal angles becomes a more balanced function of the growth. The retardance R distribution exhibits the same trend as the D distribution, with an average value of 5° at 4 hpf, while remaining constant after 72 hpf, at which point the distribution spreads moderately to larger values at 25°. This behavior confirms the loss of compactness at 4 hpf and the structural uniformity for the next stages. The distribution of the azimuthal retardance orientation α_R_ offers higher contrast, ranging from 0° at 4 hpf to −15° at 72 hpf, with a wider distribution for this last stage explained by strongest variability of shapes and structures between the different 72 hpf larvae. It is worth noting that the standard deviation due to the biological complexity of the zebrafish and the variation of the polarimetric parameters depends strongly on (1) the thickness of the sample, (2) its orientation related to the laboratory axis and (3) the focal plane position.

### Polarimetric imaging of zebrafish tail

**Figure 4:**
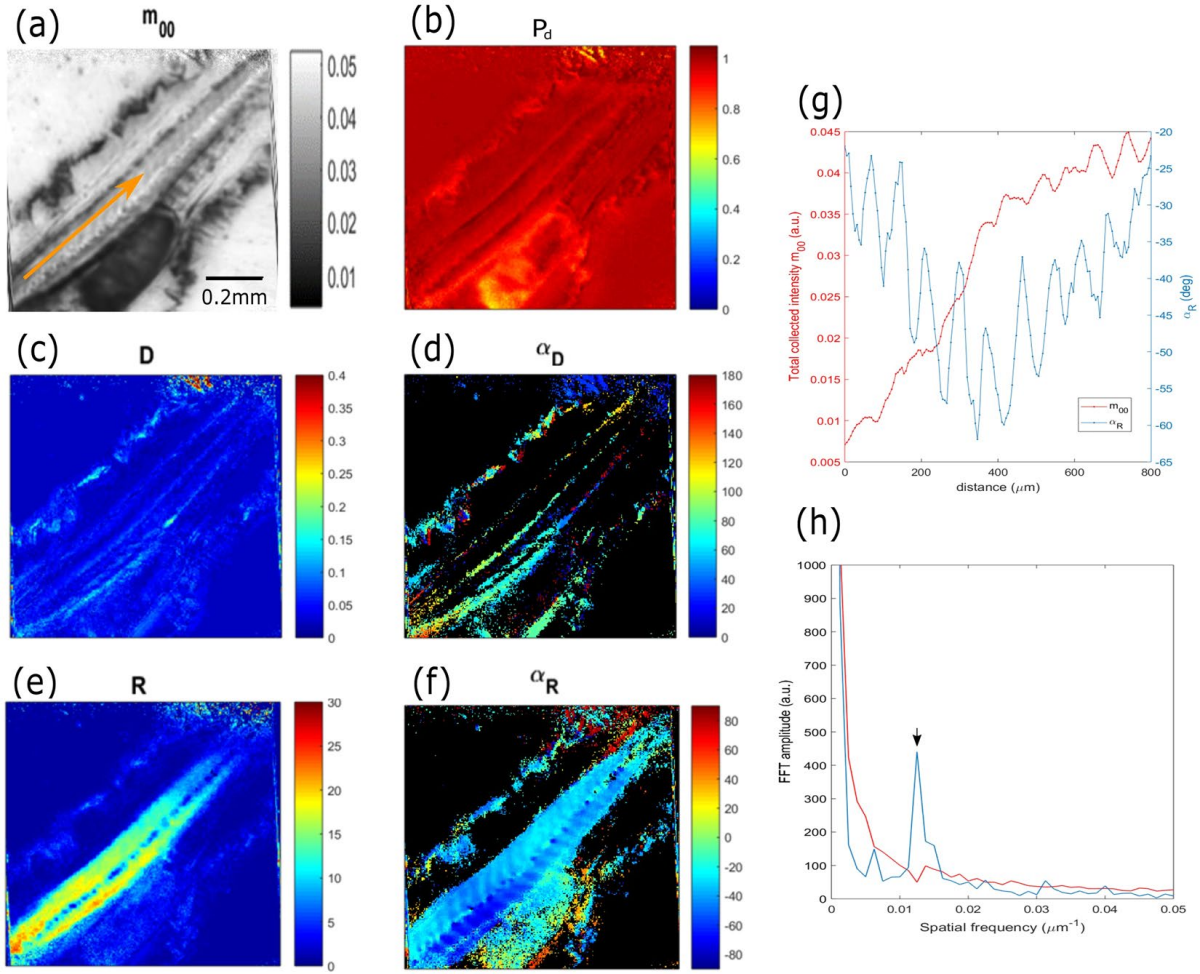
(**a**) m_00_ image of fixed larvae tail at 72 hpf for a 10X/0.4NA objective zoom 1.1. (**b–f**) Polarimetric images from the Lu and Chipman decomposition coded in (**b**) depolarization index P_d_, (**c**) diattenuation D and (**d**) its orientation α_D_, (**e**) retardance R and (**f**) its orientation α_R_. (**g**) Intensity profiles from the orange arrows in Figures (**a,e**), where the red and blue lines correspond to the m_00_ and the α_R_ intensity, respectively. (**h**) 1D Fast Fourier Transform (FFT) of the intensity profiles of (**g**).

In zebrafish larvae, the tail is composed mainly of fast and slow muscle fibers positioned at 45° related to the central bone. The function mechanisms are also different; the fast muscle fibers, located the deeper portion of the body, are used for short and brief movements, while the slow muscle fibers, located in a wedge-shaped region between the skin and the cartilage, are used for stability of the animal^33^. It has been proved that this higher-ordered periodical distribution of myosin filaments induces a strong birefringent signal^34^ giving access to the orientation of this striated muscular tissue. Therefore, it constitutes an archetypical part of the organism by which to quantify our polarimetric label-free contrasts, containing numerous structures, ranging from macroscopic to microscopic features. Figure 4 shows the m_00_ and the polarimetric images from Lu and Chipman decomposition of the larvae tail at 72 hpf using a 10X/0.4NA objective. The m_00_ and P_d_ images Fig. 4(a),(b) represent the total loss of light through the whole sample due to the absorption and scattering processes, respectively, and exhibit the same thickness differential mapping of the tail. Indeed, because of the transparency of this thin region of the zebrafish, the main optical process is mainly due to the scattering with a weak absorption effect. For this reason, the yolk sac presents more depolarization (P_d_ < 1) than the other thinner structures of the zebrafish. The images Fig. 4(c),(d), coded in D and in α_D_, highlight specifically the presence of the blood vessels and the cartilage organization, while the images Fig. 4(e),(f) coded in R and in α_R_ can be used to observe the muscular structures. Analyzing the α_R_ image Fig. 4(f) compared to the m_00_ image Fig. 4(a), the contrast of the tail was improved and each slow muscle unit could be seen. Indeed, by plotting the intensity profiles Fig. 4(g) from the orange line profile of these two images, it was proven that the m_00_ parameter was not enough to distinguish the structures of the tail muscles, while the α_R_ image allowed the quantifying of its dimension using an imaging system of the same resolution. In this way, the space between each maximum corresponding to the position of the muscle unit taken on eight slow muscles gave the value of (77.66 ± 8.16) μm, an almost similar dimension to the value found in the literature^35^. By performing a 1D Fast Fourier Transform (FFT) of the intensity plot profile in Fig. 4(g), the measurement of the spatial frequency with the highest non-DC FFT amplitude (black arrow Fig. 4(h)) corresponded to the value 0.0125 μm^−1^ which is equal to 80 μm in good agreement with our previous measurement.

**Figure 5:**
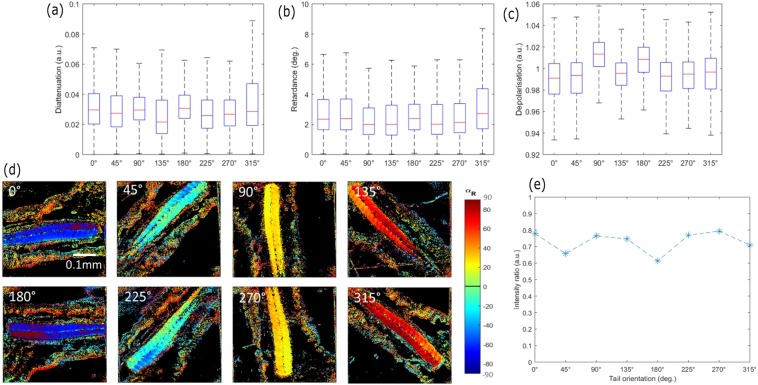
Lu and Chipman decomposition images of the fixed zebrafish tail at 72 hpf presented Fig. 4 for different orientations in the sample plane. (**a**) Diattenuation, (**b**) retardance and (**c**) depolarization index pixel distribution over the full image for each orientation. (**d**) α_R_ images of the same tail Fig. 4 at different orientations related to the sample plane. (**e**) Intensity ratio, between the maximum and minimum intensity of the muscular tissue region from the tail for the α_R_ images in (**d**).

The advantage of using Mueller-matrix microscopy is the ability to measure the orientation without loss of contrast, as has been widely demonstrated for materials characterization in ellipsometry^36,37^, contrary to other polarization-based microscopy techniques. Indeed, Mueller-matrix polarimetry is based on generating and analyzing a set of 16 independent polarization states, which amounts to no consideration for any preferential orientation of the sample under illumination. In order to evaluate the SNR in the function of the sample orientation, Fig. 5 presents the polarimetric parameters extracted from the Lu and Chipman decomposition of a larvae tail from Fig. 4, imaged at different orientations related to the sample plane.

Figure 5(a)–(c) present respectively the diattenuation, retardance and depolarization images and Fig. 5(d) presents the α_R_ images for each orientation. To evaluate the SNR by rotating the sample, Fig. 5(e) presents the ratio I_max_/I_min_ for each α_R_ orientation images in the muscular tissue region from the tail. It can be observed that the value dispersions for each polarimetric parameter are similar, indicating a preservation of the contrast of the Lu and Chipman for each sample rotation angle. Furthermore, Fig. 5(e) shows that the α_R_ images present no loss of signal for the muscular tissue, and the pseudo-colors change accordingly to the orientation of the tail, spaced by 45° in the same way that the sample. It is worth noting that the fluctuation of the contrast ratio comes from the position of the focal plane through the sample contributing to increase the signal locally.

**Figure 6:**
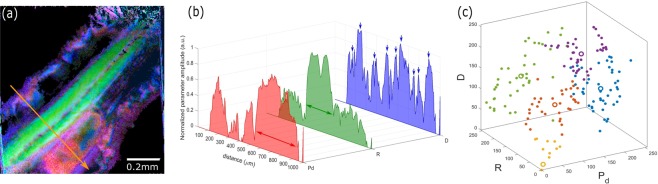
(**a**) Polarimetric merged images of the fixed zebrafish tail at 72hpf from Fig. 4 coded in depolarization index (red), retardance (green) and diattenuation (blue). (**b**) Merged normalized intensity profiles of the orange arrow in (**a**) from the three channels, i.e. depolarization index P_d_ (red), retardance R (green) and diattenuation D (blue). The green, red and blue arrows showed the estimated muscular, yolk sac and the membranes areas, respectively. (**c**) 3D-clustered pixels intensity distribution of the three main polarimetric parameters from Fig. 4(b),(c),(e).

To improve the visualization of the specific fingerprints of each biological structure of the zebrafish, we propose, in Fig. 6, a merged polarimetric image of the three main parameters from the Lu and Chipman decomposition shown in Fig. 4.

Figure 6(a) highlights the capability of Mueller-matrix imaging to discriminate the specific localized regions of a tail in a label-free way at the single microscopic level since it was possible to obtain with fluorescence labeling. Figure 6(b) shows the intensity profiles for the three polarimetric channels from the orange arrow in Fig. 6(a) to identify the different structures of the larvae tail at 72hpf. Using the P_d_ and R intensity plots, the estimated yolk sac and the muscular region were identified separately (area of maximum amplitudes) with the red and green arrows, respectively, while the blue arrows indicated the presence of membrane structures. Figure 6(c) shows the pixel values distribution from the intensity profile of the three polarimetric parameters. This representation clearly highlights five-pixel clusters, which localized the maximum contrast from each channel. In accordance with the description above, it is worthy to note that this additional quantitative tool, widely used for single molecule localization microscopy, is an interesting possibility to cross-correlate every zebrafish structure with the physical imaging process.

## Discussion

Our Mueller-matrix microscope in reflection configuration enables us to acquire a detailed fingerprint of fixed zebrafish at different developmental stages. This is key to gain an in-depth understanding of the morphological changes of the animal during development. Because each biological structure exhibits a specific physical response to light, the different parts of the zebrafish can be discriminated using appropriate encoding polarimetric values. We believe that this convenient, easy and inexpensive label-free method gives access to localized tracking of the fingerprint changes of the biological organization without a priori knowledge of the sample. Furthermore, zebrafish live imaging could be possible to perform by embeding the embryos in agarose gel to prevent movement, as in light-sheet microscopy, which is not significantly different from the post-mortem samples used in this study. However, given the success of these initial results, we plan in the future to extend our studies to live zebrafish embryos. The technique is compatible with fluorescence microscopy as well as non-linear imaging techniques, which can help to develop correlative approaches to obtain an unprecedented characterization of a biological specimen. The non-invasive and label-free nature of Mueller-matrix microscopy can pave the way for studying the polarimetric changes in animal models in the presence of pathologies, such as myopathy, thus helping the advance towards easy and cheap biomedical diagnosis.

## Methods

### Experimental setup

**Figure 7:**
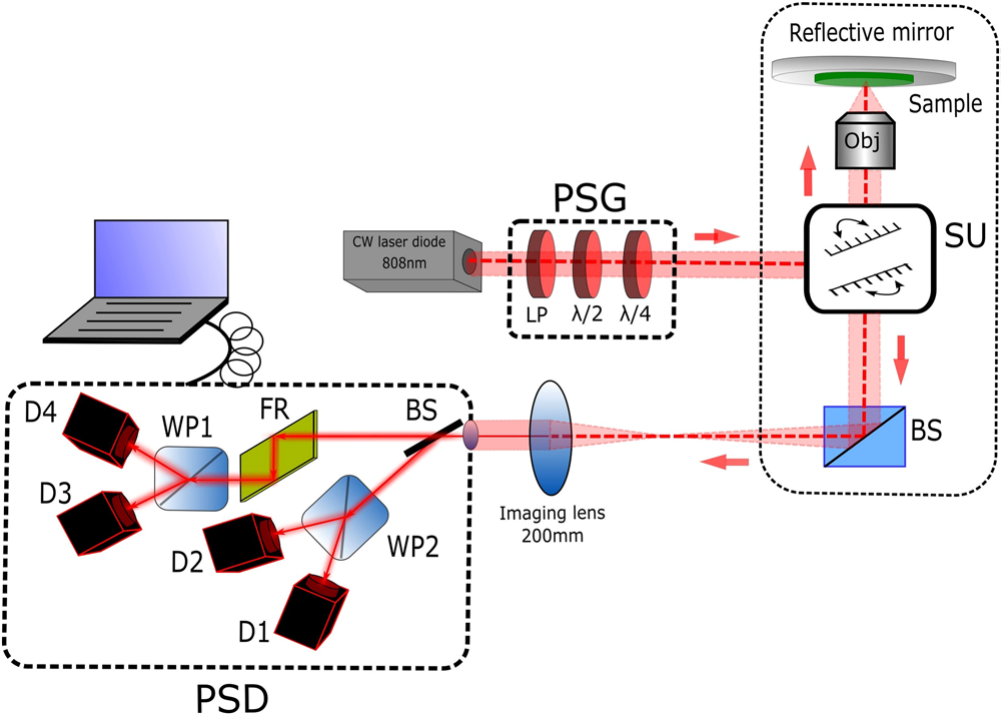
Schematic diagram of the experimental setup in reflection configuration. PSG: Polarization States Generator. PSD: Polarization States Detection. LP: Linear Polarizer. λ/2: Half-waveplate. λ/4: Quarter-waveplate. SU: Scanning Unit. BS: Beamsplitter. Obj: Objective lens. FR: Fresnel Rhomb. WPi (I = 1, 2): Wollaston Prism. Di (i = 1, 2, 3, 4): Detector.

The experimental setup was a modified inverted commercial confocal microscope Olympus FV1000 described in detail in Fig. 7.

The light source was a tunable intensity CW laser diode at 808 nm (MLL-III-808–2.2 W, CNILASER) delivering 1 mW at the sample plane. This laser power is arbitrarily fixed for all the measurements since, in Mueller-matrix microscopy, the excitation power is not a limitation if the SNR is high enough at the detection plane. A Polarization States Generator (PSG) was placed as input of the microscope body. The PSG consisted of a Linear Polarizer (LP; 25.4 mm diameter, VISIR CW02, Newport), a Half-Wave Plate (HWP, λ/2; 12.7 mm diameter, Thorlabs, SAHWP05M-700, Inc., USA) and Quarter-Wave Plate (QWP, λ/4; 12.7 mm diameter, Thorlabs, SAQWP05M-700, Inc., USA). The optical elements were placed into a motorized rotating stage (PR50CC, Newport), controlled by a stepper motion controller (ESP3013-Axis DC, Newport). In this way, the PSG generated sequentially four input polarization states 0° (H), 90° (V), 45° and right circular polarization (RCP) corresponding to the Stokes vectors (1 1 0 0), (1–1 0 0), (1 0 1 0) and (1 0 0 1). To image the thin sample in reflection with the laser scanning unit (SU; FV1000, Olympus, Japan), we placed a silver plane reflective mirror (Thorlabs, PF10-03-P01, Inc., USA) at the focal plane of the low numerical aperture objectives used in this study to prevent all the negative effect of the polarimetric parameters. Here, we used a 4X/0.1NA (Plan Achromat; Olympus, Japan) and 10X/0.4NA (UPLSAPO; Olympus, Japan) objectives, which correspond to an optical resolution R ≈ λ/(2.NA) of ≈ 4 μm and ≈ 1.1 μm respectively, and field of view (FOV) of 1.5 × 1.5 mm² and 0.9 × 0.9 mm² respectively. In this study, the confocal mode of the microscope is not used, which required complex additional calibration steps, as has been proven in early works^29^. The results presented correspond to the polarimetric fingerprint averaged in all the PSF volume. The image of the scanning beam plane was collected through a 200 mm focal length imaging lens (Thorlabs, AC508-200-B-ML, BBAR coating 650–1050 nm, Inc., USA) at the exit of the microscope body. The passive four-channel Polarization States Detection (PSD) analyzed the transformation of the polarization after the interaction of the polarized light with the sample, based on the reference^38,39^. It consisted of a thin glass slice oriented at 75°, a Fresnel rhomb (FR; Thorlabs, FR600 QM, Inc., USA) and two Wollaston prisms (WP1 and WP2; Thorlabs,WP10, Inc., USA) and four PDAs (D1, D2, D3 and D4; Thorlabs, Det36A2, 350–1100 nm, Inc., USA) to collect the signal pixel by pixel for each four-polarization states. The four-input polarization states generation and the four collected images were synchronized with the scanning unit using a LabVIEW 2017 (National Instrument, Austin, Texas) homemade routine via a data board acquisition (DAQ, National Instrument, Austin, Texas). Finally, a Matlab program (Matlab, v.R2017a, Mathworks) was used to extract the full Mueller-matrix of the sample pixel-by-pixel from the acquisition of the 16 collected images (four images by input polarization state). To synchronize properly the LabVIEW program with the scanning system due to the DAQ delay time, the pixel dwell time was set arbitrary at 100 μs per pixel, which resulted in a total acquisition time of around 30 s for a full Mueller-matrix image. In instances where SNR was poor, we performed frame averaging (5 frames, for a total acquisition time of about 3 min). Because of the slow dynamics of current fixed samples, higher temporal resolutions are not required. In any case, the sequential measurement can be speeded up by replacing the rotation motor of the optical components from the PSG by liquid crystal variable retarders or electro-optics devices^40^, or by spectrally encode/decode the polarization states at a high-speed rate, as proposed in recent works^28,30^.

### Calibration steps

The calibration procedure consisted of two steps to take into account the remaining optical orientation misalignment of the PSG and PSA, as well as the polarimetric signature of the optical devices of the microscope body, composed mainly by lenses, reflective mirrors, and scanners. First, to calibrate the PSG and PSA, the Eigenvalue Calibration Method was used in air, with a LP and a QWP (similar to the ones used for the PSG) as reference samples^41–43^. In this case, the system was judged based on the condition number (CN) of the instrument matrix; the lower the value of CN, the better is the accuracy of the polarimeter. In this case, we placed the PSG after the scanning unit, to achieve a CN of 2.6. Next, we placed the PSG before the microscope to image the Mueller-matrix of the whole system [M_μ_ (x, y)] taking into account the polarimetric fingerprint of the microscope pixel-by-pixel in the entire field of view, which is mainly composed of the dephasing effect on the polarization^28^. In the backscattering configuration, the optical path through the microscope was separated into two Mueller-matrices, named $[{{\rm{M}}}_{{\rm{\mu }}}^{{\rm{b}}}({\rm{x}},{\rm{y}})]$ and $[{{\rm{M}}}_{{\rm{\mu }}}^{{\rm{a}}}({\rm{x}},{\rm{y}})]$, to take into account the optical signature before and after the sample, respectively. Therefore, the Mueller-matrix of the sample [M_sample_ (x, y)] was extracted from the total measurement [M_mes_ (x, y)] by applying the following equation:

1 $$[{{\rm{M}}}_{{\rm{sample}}}({\rm{x}},{\rm{y}})]={[{{\rm{M}}}_{{\rm{\mu }}}^{{\rm{a}}}({\rm{x}},{\rm{y}})]}^{-1}.[{{\rm{M}}}_{{\rm{mes}}}({\rm{x}},{\rm{y}})].{[{{\rm{M}}}_{{\rm{\mu }}}^{{\rm{b}}}({\rm{x}},{\rm{y}})]}^{-1}$$

Considering that after the sample, light passed only through a non-polarized beam splitter, the polarimetric changes in this part could be neglected, which yields to:

2 $$[{{\rm{M}}}_{{\rm{\mu }}}^{{\rm{b}}}({\rm{x}},{\rm{y}})]\approx [{{\rm{M}}}_{{\rm{\mu }}}({\rm{x}},{\rm{y}})]$$

3 $$[{{\rm{M}}}_{{\rm{\mu }}}^{{\rm{a}}}({\rm{x}},{\rm{y}})]\approx {{\rm{I}}}_{4}$$

where I_4_ is the 4 × 4 identity diagonal matrix. Thus, the sample Mueller-matrix could be written as:

4 $$[{{\rm{M}}}_{{\rm{sample}}}({\rm{x}},{\rm{y}})]\approx [{{\rm{M}}}_{{\rm{mes}}}({\rm{x}},{\rm{y}})]\,\cdot \,{[{{\rm{M}}}_{{\rm{\mu }}}({\rm{x}},{\rm{y}})]}^{-1}$$

In practice, this approximation means that the polarimetric signature could be retrieved in a simple two-step process. First, acquisition of the microscope fingerprint in reflection with the mirror alone. Second, acquisition of the polarimetric signature of both sample and microscope. Thereby, the Mueller-matrix image of the sample was obtained by inverting directly the microscope fingerprint. Equation (4) does not consider the π-dephasing at the reflection of the mirror surface, but we observed that it was removed after the reflection with the beam-splitter cube in the backward optical path to the PSD^44^. Importantly, the double pass through the biological sample improves the polarimetric signal and the imaging contrast, which are intrinsically weak in biological samples. Note that the phase value φ_measured_ from the double pass optical path is defined as modulo 2π, so the exact value φ_exact_ was recovered by applying φ_exact_ = 2π − φ_measured_.

### Lu and Chipman decomposition

To separately extract each polarimetric component under study in this paper, we implemented a Lu and Chipman decomposition algorithm^11,45^. It is based on the decomposition of the interaction between the sample and the polarized light into the three main optical interactions; the scattering, birefringence and dichroism, as a product of the three matrices associated to the polarimetric parameters:

5 $${{\rm{M}}}_{{\rm{sample}}}={{\rm{M}}}_{\Delta }\,\cdot \,{{\rm{M}}}_{{\rm{R}}}\,\cdot \,{{\rm{M}}}_{{\rm{D}}}$$

where M_Δ_, M_R_ and M_D_ are the sample matrices describing the depolarization, the linear and circular birefringence, and the linear and circular birefringence, respectively. After normalization by the m_00_ element, the decomposition gives access to the physical parameters denoted in this paper by:

6 $${{\rm{P}}}_{{\rm{d}}}=\sqrt{\frac{{\sum }_{{\rm{i}},{\rm{j}}=0}^{3}{{\rm{m}}}_{{\rm{ij}}}^{2}-{{\rm{m}}}_{00}^{2}}{3{{\rm{m}}}_{00}^{2}}}$$

7 $${\rm{R}}={\rm{arcos}}(\frac{{\rm{tr}}({{\rm{M}}}_{{\rm{R}}})}{2}-1)$$

8 $${\rm{D}}=\sqrt{{{\rm{m}}}_{01}^{2}+{{\rm{m}}}_{02}^{2}+{{\rm{m}}}_{03}^{2}}$$

where, P_d_, R and D correspond to the depolarization index, retardance and diattenuation, m_ij_ are the Mueller-matrix elements and tr(M_R_) is the trace of the retardance matrix M_R_. In this way, each of these three physical parameters lies between 0 ≤ P_d_ ≤ 1, 0° ≤ R ≤ 180° and 0 ≤ D ≤ 1, and is used for coding pixel-by-pixel the post-treated images. It is worth noting that the amplitudes of these three parameters are directly related to the sample properties.

### Validation on reference samples

Images of reference samples in reflection configuration were acquired to validate the calibration procedure defined above and the accuracy of the Mueller-matrix images. First, the statistical data on each polarimetric parameters without sample for the 4X/0.1NA were measured for D = 0.025 ± 0.012, R = 2.00° ± 0.92° and P_d_ = 1.003 ± 0.019. Table 1 shows the measured polarimetric parameters of 256 × 256 pixels images of thin reference samples ( < 1 mm thickness) which presented mostly dichroism (in theory, D = 1, R = 0, P_d = _1) and birefringence (in theory, D = 0, R ≠ 0, P_d = _1), such as a LP (LP; VISIR CW02, Newport) and a single layer cellophane tape.

**Table 1:** Polarimetric parameters of the linear Diattenuation (D), linear Retardance (R), Depolarization index (P_d_) and its orientations (α_D_ and α_R_) measured for a Linear Polarizer (LP) and for a cellophane tape surface (Tape) at 808 nm.

		D	α_D_	R	α_R_	P_d_
4X/0.1NA, 1.5 × 1.5 mm²	LP	0.970 ± 0.023	94° ± 2°	X	X	1.074 ± 0.070
	Tape	0.067 ± 0.020	X	14° ± 1°	−18° ± 3°	1.012 ± 0.021
10X/0.4NA, 0.9 × 0.9 mm²	LP	0.989 ± 0.020	126° ± 2°	X	X	0.943 ± 0.057
	Tape	0.075 ± 0.020	X	17° ± 1°	−10° ± 2°	1.065 ± 0.021

Values were given for the 4X/0.1NA and 10X/0.4NA objectives used. The “X” symbol meant that the parameter had no physical meaning for the corresponding reference sample.

These data show accurate measurements in every polarimetric parameters thus justifying the use of the microscope for characterizing biological structures. Notably, this statistical error within the whole field of view depends on the optical misalignment, the PSG/PSA matrix evaluation and the sample surface inhomogeneity. Particularly, the depolarization is a mixed between the scattering effects coming from the specific fingerprint of the biological structures under illumination, such as the thicker part of the zebrafish (almost composed by the yolk sac) and experimental errors. Fortunately, Lu and Chipman decomposition allows to extract separately the depolarization induced by scattering, and the remaining errors on the Mueller-matrix elements. Still, statistical errors could be further reduced by using, for instance, the spectral decomposition based on the interpretation of the eigenvalues of the Mueller-matrix^46,47^. This accuracy could be improved by reaching the theoretical value of CN = 1.77^48^ or by using more sensitive photodetectors.

#### Sample preparation

Wild-type adult zebrafish (*Danio Rerio*) were maintained under conditions previously described^49^. Healthy embryos at 4 hpf were collected and maintained in embryo medium in an incubator at 28 °C^50^. Embryos and larvae at 4 hpf, 24 hpf, 48 hpf and 72 hpf were selected for imaging. Optical images were collected on Nikon SMZ18 Stereomicroscope (Nikon, Tokyo, Japan) attached to a CCD camera. Subsequently, zebrafish were fixed overnight in 4% paraformaldehyde (PFA) in phosphate buffered saline (PBS) (0.1 M, pH 7.4). After fixation zebrafish embryos and larvae were washed three times with PBS (0.1 M, pH 7.4) and immobilized on a cover slip with 0.5% agarose gel in embryo medium to avoid artifacts due to the cellular degradation. All animal experiments were performed in full compliance with the revised directive 2010/63/EU.

#### Image processing

To appreciate the different area of the images Fig. 2 is coded in polarimetric orientation αD and αR, a mathematical filter was applied to remove the background for the zero value of D and R. It was fixed at a NaN values when the polarimetric parameter value was below the standard deviation. Some residual noisy pixels were not removed, probably because of an inhomogeneous field of view, specifically with the vignetting effects around the corner part of the image. To merge the three main polarimetric images Fig. 6 coded in D (blue), R (green) and P_d_ (red), the imaging data has been (1) converted into 8-bits format, (2) noisy filtered and (3) normalized by the maximum pixel intensity. To compare the same background trend for each polarimetric channel, the original P_d_ image in Fig. 4(b) was replaced by the depolarization factor, widely noted Δ^51–53^, coded between 0 for a non-depolarizing and 1 for a depolarizing medium pixel-by-pixel.

## Data Availability

The datasets generated and/or analyzed in the current paper are available from the corresponding author on reasonable request.
